# Dr. Squibb

**Published:** 1882-03-20

**Authors:** 


DR. SQUIBB is issuing a pamphlet entitled an "Ephemeris of Materia Medica, Pharmacy, Therapeutics and Collateral Information." We have the second number. Others are to be issued irregularly as may suit the author's convenience. Although gratuitously distributed, they contain matter of interest and instruction to the profession.

